# Inhibition Mechanism, Multi-Target Regulation, and Protective Effects of Camel Casein ACE-Inhibitory Peptide on HUVECs Cells

**DOI:** 10.3390/nu18091436

**Published:** 2026-04-30

**Authors:** Fei Zhang, Hao Miao, Chenkun Huo, Ruiqi He, Yanan Qin, Jie Yang, Zhongkai Zhao

**Affiliations:** 1 College of Smart Agriculture (Research Institute), Xinjiang University, Urumqi 830017, China; 13856123842@163.com (F.Z.); mhvip98@163.com (H.M.); 18230115768@163.com (C.H.); ruiqi_h@163.com (R.H.); 2 Xinjiang Key Laboratory of Biological Resources and Genetic Engineering, College of Life Science & Technology, Urumqi 830017, China; qin@xju.edu.cn (Y.Q.); yangjie234@xju.edu.cn (J.Y.); 3 Xinjiang Camel Industry Engineering Technology Research Center, Urumqi 830046, China

**Keywords:** camel casein, ACE-inhibitory peptides, molecular docking, network pharmacology, HUVECs cells

## Abstract

Hypertension is a severe global public health issue. Food-derived angiotensin-converting enzyme (ACE)-inhibitory peptides have shown great potential as safe and effective alternatives to synthetic antihypertensive drugs. Camel milk is rich in bioactive peptides. This study aimed to screen for ACE-inhibitory peptides from hydrolyzed camel casein, explore their inhibitory mechanisms and endothelial protective effects in vitro, and reveal their potential antihypertensive pathways using network pharmacology. This study screened three peptides with angiotensin-converting enzyme (ACE) inhibitory activity from enzymatically hydrolyzed camel casein components: MVPFLQPK, VPFLQPKVM, and QKWKFL, with IC_50_ values of 277.1, 396.9, and 486.9 μmol/L, respectively. Enzyme inhibition kinetics analysis indicated that MVPFLQPK exhibited a non-competitive inhibition pattern, VPFLQPKVM exhibited a mixed inhibition pattern, and QKWKFL exhibited a competitive inhibition pattern. Molecular docking revealed that all three peptides formed hydrogen bond interactions with ACE, and QKWKFL and VPFLQPKVM directly bound to the enzyme’s active site to inhibit substrate catalysis. Molecular dynamics simulation further confirmed the high stability of the three peptide–ACE complexes, with binding free energies from −34.24 to −51.19 kcal/mol. The primary contributing forces include hydrogen bonds, van der Waals interactions, electrostatic forces, and nonpolar solvation effects. Network pharmacology analysis suggested that these peptides may exert synergistic antihypertensive effects by regulating multiple blood pressure-related pathways, including the renin–angiotensin system, renin secretion, and calcium signaling pathways, by acting on key targets such as ACE, REN, SRC, and MMP9. Cell experiments demonstrated that all three peptides exhibited no cytotoxicity in the Ang II-induced HUVEC injury model, significantly promoted NO release, inhibited ET-1 secretion, and possessed endothelial protective potential. This study investigated the in vitro ACE-inhibitory mechanism of peptides derived from camel milk and their potential role in blood pressure regulation, providing experimental evidence for subsequent in vivo activity validation and the development of functional camel milk protein products.

## 1. Introduction

Hypertension is a symptom of cardiovascular disease, and it is one of the leading causes of premature death from cardiovascular-related conditions worldwide [1]. The World Health Organization (WHO) ranks hypertension as the third leading cause of reduced life expectancy due to physical disabilities. According to the Global Hypertension Report, the number of people with hypertension worldwide has surged significantly over the past 30 years, increasing from 650 million to 1.28 billion [2]. It is evident that combating hypertension is a global health challenge. Currently, hypertension management primarily involves lifestyle intervention and clinical drug therapy. Pharmacological treatment mainly involves the use of diuretics, beta-blockers, angiotensin-converting enzyme (ACE) inhibitors, angiotensin receptor blockers, and calcium channel blockers to regulate blood pressure. However, patients with hypertension often experience multiple symptoms, necessitating the use of various medications for treatment, which may lead to adverse drug reactions [3]. Furthermore, hypertension frequently causes complications that reduce the efficacy of medications and adversely affect metabolism. Consequently, consumers are increasingly inclined to use natural food-derived peptides to regulate blood pressure through dietary interventions, aiming to reduce drug-related damage [4].

Blood pressure regulation in the human body primarily involves two systems: the renin–angiotensin system (RAS) and the kallikrein–kinin system (KKS). Elevated blood pressure is mainly driven by the RAS, whereas vasodepressor effects are primarily attributed to the KKS. A balanced interaction between these two systems is critical for maintaining normal blood pressure homeostasis. Angiotensin-converting enzyme (ACE, EC 3.4.15.1), a zinc metallopeptidase, plays a central role in both systems and regulates blood pressure. In the RAS, reduced renal perfusion pressure stimulates renin secretion, which converts angiotensinogen into inactive angiotensin I (Ang I). ACE then catalyzes the conversion of Ang I into angiotensin II (Ang II), a potent vasoconstrictor. Ang II induces vasoconstriction, inflammation, and oxidative stress, thereby increasing blood pressure [5]. In the KKS, kallikrein cleaves kininogen to release bradykinin, a potent vasodilator that lowers the blood pressure. ACE is also responsible for the rapid degradation and inactivation of bradykinin [6]. Excessive ACE activity disrupts the physiological balance between RAS and KKS, leading to dysregulation of blood pressure and the development of hypertension. Therefore, inhibiting ACE activity is an effective strategy for managing hypertension. Although synthetic ACE inhibitors are widely used clinically, long-term administration is often accompanied by adverse side effects [7]. Food-derived ACE-inhibitory peptides have attracted increasing attention owing to their high safety, good biocompatibility, and targeted inhibitory effects, making them promising candidates for hypertension intervention.

Animal milk proteins have long served as an excellent source of ACE-inhibitory peptides [8]. Among these, camel milk is a highly nutritious dairy product rich in proteins, vitamins, fats, minerals, and various natural bioactive components, such as lactoferrin, lysozyme, and immunoglobulins [9]. Although its chemical composition resembles that of cow and sheep milk, camel milk exhibits higher concentrations of these nutrients [10,11]. Research indicates that camel milk effectively treats hypertension, hyperlipidemia, diabetes and its complications, kidney disease, immune deficiency, depression, and cancer [12], which helps maintain cardiovascular health [13]. Moreover, these bioactive components can be produced in significant quantities during fermentation and enzymatic hydrolysis. Progress has been made in extracting ACE-inhibitory peptides from camel milk protein. Through methods such as enzymatic hydrolysis and fermentation, bioactive peptides have been isolated from whole milk proteins, whey proteins, casein, and lactoferrin. In a similar study, Nazila Soleymanzadeh et al. [14] identified a novel polypeptide MVPYPQR with antioxidant activity (8933.05 μM TE mg^−1^) and ACE-inhibitory activity (IC_50_ = 30 μM) from fermented camel milk. Moreover, Mainasara et al. [15] studied the therapeutic effects of camel milk on hypertension. The results showed that the systolic blood pressure of rats in the experimental group drinking camel milk decreased significantly, indicating that camel milk had an obvious improvement effect on salt-induced hypertension. In conclusion, the active polypeptides and their fermentation products in camel milk exhibit significant antihypertensive potential. However, the current studies on the structure–activity relationship of active peptides are still limited.

Based on our previous work, three ACE-inhibitory peptides—QKWKFL, VPFLQPKVM, and MVPFLQPK—were identified through the enzymatic hydrolysate components. In this study, these peptides underwent solid-phase synthesis, and their half-maximal inhibitory concentrations and inhibition patterns were determined. Meanwhile, preliminary exploration of the potential inhibitory mechanisms of these three ACE-inhibitory peptides was conducted using molecular docking, molecular dynamics simulations, and network pharmacology. Furthermore, an angiotensin II (Ang II)-induced HUVEC injury model was established to investigate the protective effects of the peptides on damaged HUVECs. The effects of peptide on intracellular regulatory factors such as NO and ET-1 were also measured. This study aimed to explore the potential mechanisms and in vitro biological activities of peptides derived from camel milk casein in the adjunctive regulation of hypertension and to provide experimental references for the subsequent research and development of functional camel milk products related to blood pressure regulation.

## 2. Materials and Methods

### 2.1. Materials

Peptides were synthesized by Gen Script Biotech Corp. (Piscataway, NJ, USA) with a purity of ≥95%. Primary human umbilical vein endothelial cells (HUVEC) were purchased from Scien Cell Research Laboratories (Carlsbad, CA, USA). Hippuric acid (standard) ≥98% (HPLC) was purchased from Beijing Sola Bio Technology Co., Ltd. (Beijing, China); pony uric acid histidyl-L-leucine ≥98% was purchased from Beijing Sola Bio Technology Co., Ltd.; angiotensin-converting enzyme 0.1 U was purchased from Sigma-Aldrich, (St. Louis, MO, USA); angiotensin II was purchased from Med Chem Express (Monmouth Junction, NJ, USA); ECM medium was purchased from Scien Cell Research Laboratories, (Carlsbad, CA, USA); the nitric oxide assay kit was purchased from Wuhan Elabrite Biotechnology Co., Ltd. (Wuhan, China); the human endothelin-1 (ET-1) quantitative ELISA assay kit was purchased from Nanjing Jian Cheng Bioengineering Institute (Nanjing, China); the 0.22 μm microporous filter membrane was purchased from Merck Millipore (Billerica, MA, USA); the 96-well plate was purchased from Corning (Corning, NY, USA).

### 2.2. ACE Inhibition Assay

The concentrations of Hippuryl-His-Leu (HHL) and its hydrolyzed product Hippuric Acid (HA) were measured using an Agilent 1260 Infinity II HPLC system and an Agilent C18 column (150 × 4.6 mm, 5 μm particle size). Mobile phase A was an aqueous solution containing 0.05% trifluoroacetic acid (TFA), and mobile phase B was acetonitrile (ACN). Isocratic elution mode was employed with a mobile phase ratio of 78% A and 22% B. The flow rate was set to 0.5 mL/min, and the elution time was 25 min. UV detection was performed at 228 nm. The ACE inhibition rate (%) was calculated using the following formula:

$$\mathrm{ACE}\;\mathrm{Inhibitory}\;\mathrm{Activity}\;(\%)=\frac{\Delta A_{\mathrm{blank}}-\Delta A_{\mathrm{inhibitor}}}{\Delta A_{\mathrm{blank}}}\times 100$$

In the equation, Δ blank and Δ inhibitor represent the peak areas of HA in the blank (sodium borate buffer solution) and the sample, respectively.

### 2.3. Analysis of ACE-Inhibitor Peptide Inhibition Patterns

Based on the IC_50_ values of the three peptides, (A)peptide MVPFLQPK was set up at concentrations of 0, 150, and 300 µM; (B) peptide VPFLQPKVM was set up at concentrations of 0, 200, and 400 µM; (C) peptide QKWKFL was set up at concentrations of 0, 250, and 500 µM. All peptides were co-incubated with HHL and ACE solutions at varying concentrations. Three parallel experiments were conducted for each peptide concentration, and the average values were used to generate the Lineweaver–Burk plots. The ACE inhibition patterns of the peptides were investigated using Lineweaver–Burk plots based on the peak area curves of HHL [16].

### 2.4. Molecular Docking

The crystal structure of ACE (PDB ID: 1O86) was obtained from the RCSB Protein Data Bank (http://www.rcsb.org/, accessed on 2 March 2026). Preprocessing was performed using the Pymol (version 2.6.0) software to remove the lisinopril ligand, dehydrate the molecule, and add hydrogen atoms, yielding the ACE protein molecule. The peptide molecules were drawn using Chem Bio Draw Ultra 14.0. Subsequently, ChemBio3D Ultra 14.0 was used to minimize the energy of the peptide, yielding the spatial conformation of the peptide small molecule. Auto Dock Vina (version 1.2.6) software was used to open the processed ACE macromolecule and peptide small molecules, designating them as receptors and ligands, respectively. A docking box was set, and semi-rigid docking was selected to perform 20 docking runs. The docking binding energies were analyzed, the optimal conformation was selected from the docking results, and the results were analyzed using Pymol software and Accelrys Discovery Studio Visualizer (version 4.1) (DS Visualizer 4.1, Accelrys Software Inc. http://www.accelrys.com, accessed on 2 March 2026).

### 2.5. Molecular Dynamics Simulation

Molecular dynamics (MD) simulations were performed on the protein–ligand complexes obtained from molecular docking using GROMACS 2020 software [17,18]. Simulations were conducted at a static temperature of 300 K and standard atmospheric pressure (1 bar) using the Amber99sb-ildn force field. The solvent consisted of water molecules (Tip3p water model), with an appropriate amount of Na^+^ ions added to neutralize the total charge of the system. Energy minimization was performed using the steepest descent method, followed by 100,000 steps of equilibration in both the Isothermal and Isovolumetric Ensemble (NVT) and Equilibrium and Isothermal-Isobaric Ensemble (NPT)with a coupling constant of 0.1 ps for 100 ps. Finally, free MD simulations were performed for a total duration of 100 ns.

### 2.6. Network Pharmacology Analysis

A peptide activity target set was constructed using the Swiss Target Prediction (http://www.swisstargetprediction.ch/, accessed on 2 March 2026) and SEA (https://sea.docking.org/, accessed on 3 March 2026) databases [19]. The Gene Cards (https://www.genecards.org/, accessed on 3 March 2026) database, OMIM (https://www.omim.org/, accessed on 3 March 2026) [20], and the Dis Ge NET (https://www.disgenet.com/, accessed on 3 March 2026) [21] database were used to identify hypertension-related genes. A Venn diagram mapping peptides to hypertension-related targets [22] was created. The target database was uploaded to the STRING [23] database for protein–protein interaction analysis. Subsequently, Cytoscape 3.9.1 was used to construct a protein–protein interaction network diagram, and the Centi scape 2.2 plugin was employed for core target screening. Additionally, GO functional analysis and KEGG pathway enrichment analysis were performed using the David (https://davidbioinformatics.nih.gov/, accessed on 3 March 2026) [24] database.

### 2.7. Cell Culture

Cells were cultivated using endothelial cell medium (ECM). When the cell density reached 80–90%, the medium was aspirated, washed twice with sterile PBS, and digested with 0.05% trypsin solution. After digestion, a complete medium was added to terminate the digestion. The cells were gently pipetted to detach them completely from the flask walls and form a cell suspension. The cells were pelleted by centrifugation at a low speed. The supernatant was removed, and the cells were resuspended in fresh complete medium and passaged at a 1:3 ratio.

### 2.8. Assay for Peptide Cytotoxicity

The HUVEC suspension was adjusted to a cell concentration of 1 × 10^5^ cells/mL. A total of 100 µL was seeded per well into a 96-well plate. After 24 h of culture, the medium was changed. The blank control group was treated with 100 µL of ECM complete medium. Next, 100 µL of ECM complete medium containing ACE-inhibitory peptides (5, 10, 20, 50, 100, 250, and 500 µmol/L) was added to each sample group. Each group contained six replicates each. After 24 h of culture, 10 µL of Cell Counting Kit-8 (CCK-8) reagent was added to each well. The absorbance was measured at 450 nm using an enzyme-linked immunosorbent assay (ELISA) reader, and cell viability was calculated.

### 2.9. Establishment of an Angiotensin II (Ang II) Injury Model

The HUVEC suspension was adjusted to a concentration of 1 × 10^5^ cells/mL and seeded at 100 µL per well into a 96-well plate. After 24 h of culture, serum-free medium was added for a 12 h starvation period to synchronize cell growth. Cells were divided into a control group (complete medium) and injury groups treated with angiotensin II (0.01, 0.10, 1.00, 10.00, 20.00, 50.00 µmol/L, and 100.00 µmol/L) for 6, 12, and 24 h. Subsequently, 10 µL of CCK-8 reagent was added to each well. Cell viability was assessed by measuring absorbance at 450 nm using a microplate reader while concurrently observing morphological changes in the cells.

### 2.10. Cell Processing and Grouping

Experiments were conducted after the cells were passaged to the 3rd to 5th generations. The experimental groups were divided into: (1) normal control group; (2) angiotensin II-injured group; (3) positive control group (Captopril); and (4) low-, medium-, and high-dose treatment groups with MVPFLQPK, QKWKFL, and VPFLQPKVM peptides.

### 2.11. NO Content Determination

Based on the results of the cytotoxicity assay, peptides at different concentrations (25, 50, and 100 μmol/L) were selected for cellular experiments to measure changes in NO concentrations. The extracellular NO production was measured using the NO Detection Kit (Wuhan Elarite Biotechnology Co., Ltd., Wuhan, China) via the nitric oxide reductase assay.

$$\mathrm{NO}\;\mathrm{Content}(\mu \mathrm{mol}/L)=\frac{\Delta A530-b}{a}\times f$$

In the equation, a represents the slope of the standard curve, b represents the intercept of the standard curve, ∆A530 represents the difference between the OD value of the test well and the OD value of the blank well, and f represents the dilution factor of the sample before it is added to the detection system.

### 2.12. ET-1 Content Assay

ET-1 levels in the culture supernatant were measured using an ET-1 detection kit (Nanjing Jian Cheng Bioengineering Institute, Nanjing, China).

### 2.13. Statistical Analysis

Results were expressed as means ± SD, *p* < 0.05. Data processing and graphing were performed using Excel and Origin (version 2024) software. Experiments were conducted in triplicate. Statistical analysis was subjected to one-way analysis of variance using SPSS 26.0 software (IBM SPSS Statistics for Windows, Version 26.0, IBM Corp., Armonk, NY, USA).

## 3. Results and Discussion

### 3.1. ACE-Inhibitory Activity of Peptides

**Figure 1:**
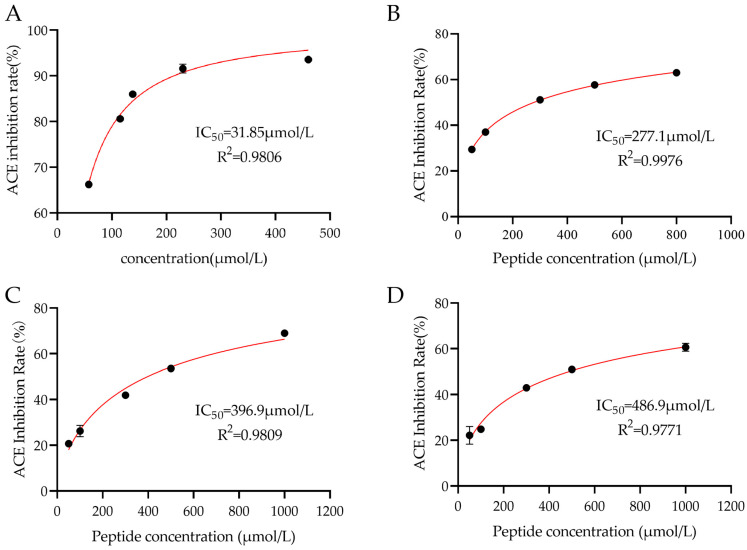
ACE half-inhibitory concentration curves of the peptides. Note: (**A**) Captopril, (**B**) peptide MVPFLQPK, (**C**) peptide VPFLQPKVM, (**D**) peptide QKWKFL.

**Figure 2:**
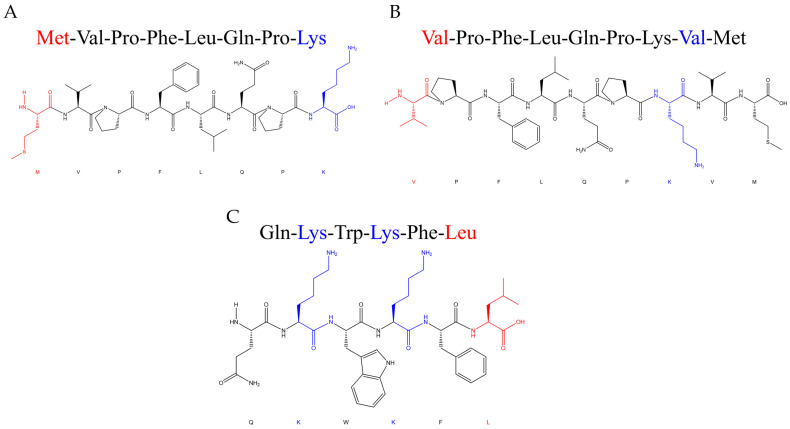
Structural formula of the peptides. Note: (**A**) peptide MVPFLQPK, (**B**) peptide VPFLQPKVM, (**C**) peptide QKWKFL.

The ACE inhibition rates of the three synthetic peptide segments are shown in Figure 1. All three peptides, QKWKFL, VPFLQPKVM, and MVPFLQPK, exhibited high ACE-inhibitory activity, with their inhibitory effects being directly proportional to the peptide concentration (Figure 2). Among them, the peptide MVPFLQPK exhibited the strongest inhibitory activity, with an IC_50_ value of 277.1 ± 0.6693 μmol/L, followed by VPFLQPKVM (396.9 ± 2.632 μmol/L) and QKWKFL (486.9 ± 2.430 μmol/L), respectively. Although the IC_50_ values of the three ACE-inhibiting peptides were higher than the positive control of captopril (31.85 ± 1.511 μmol/L), these peptides are derived from a natural casein ingredient and thus still offer certain advantages in the development of functional foods and as adjunctive treatments for hypertension.

Over the past several decades, the sequence and structural characteristics of ACE-inhibitory peptides have been identified from different food resources [25]. Generally, the short-chain peptides (2–10 amino acid residues) exhibit stronger ACE-inhibitory activity [26]. Previous studies have demonstrated that the inhibitory activity against ACE is also related to the molecular size, hydrophobicity, and structure of the peptide fragments [27,28,29]. Furthermore, the ideal model for ACE-inhibitory peptides appears to feature aromatic amino acids at the *C*-terminus, a positively charged amino acid in the middle, and hydrophobic amino acid residues at the *N*-terminus. In this study, the peptide MVPFLQPK (Figure 1A) contains multiple hydrophobic amino acids (Met, Val, Phe, and Leu), exhibiting strong hydrophobicity that facilitates binding to ACE. The peptide VPFLQPKVM (Figure 1B) also contains a diverse array of hydrophobic amino acids (Val, Phe, Leu, Val, Met) and exhibits strong hydrophobicity. However, its longer peptide chain increases steric hindrance, hindering peptide access to the ACE active site. The peptide QKWKFL (Figure 1C) possesses a shorter chain but fewer hydrophobic amino acids (only Trp, Phe, and Leu) and more hydrophilic amino acids (Gln and two Lys), resulting in overall weaker hydrophobicity that is unfavorable for binding to ACE. DALIRIEBM et al. [30] reported that positively charged amino acids (histidine H, arginine R, and lysine K) in peptide sequences may enhance the ACE-inhibitory activity of peptide fragments. Similarly, the three peptides identified in this study all contain positively charged lysine or arginine residues (Table 1), which may be a key reason for their superior ACE-inhibitory activity.

### 3.2. ACE-Inhibitor Peptide Inhibition Pattern

**Figure 3:**
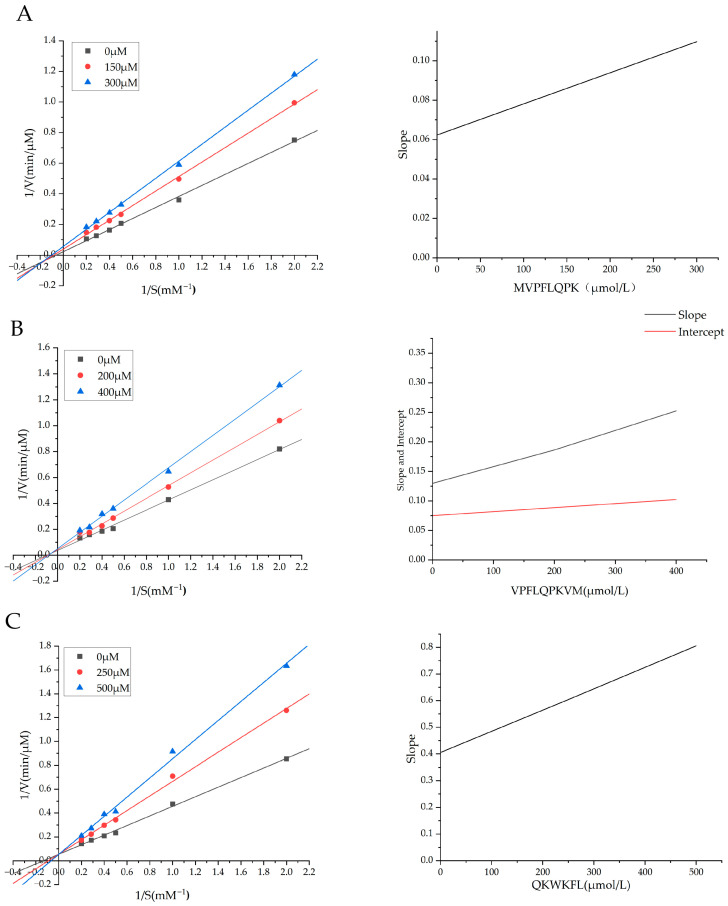
Lineweaver–Burk plots and inhibition constant secondary plots of the three peptides (**A**) MVPFLQPK, (**B**) VPFLQPKVM, (**C**) QKWKFL inhibiting ACE.

In order to evaluate the mechanisms of action of the three ACE-inhibitory peptides obtained—MVPFLQPK, VPFLQPKVM, and QKWKFL—the Lineweaver–Burk plot method was employed to investigate the inhibition patterns of these three synthetic peptides across six different concentrations of HHL. As shown in Figure 3, with the concentrations of the three peptides MVPFLQPK, VPFLQPKVM, and QKWKFL increased, specifically, the V_max_ and K_m_ of the peptide MVPFLQPK (Figure 3A) both decreased in a concentration-dependent manner, and the Lineweaver–Burk plot fell in the third quadrant, consistent with the characteristics of uncompetitive inhibition; for the peptide VPFLQPKVM (Figure 3B), as the concentration increased, V_max_ decreased, Km increased, and the double-reciprocal curve intersected in the second quadrant, indicating a mixed inhibition pattern; for the peptide QKWKFL (Figure 3C), as the concentration increased, V_max_ constantly remained, K_m_ increased, and the double-reciprocal curve intersected in the upper half of the vertical axis, indicating competitive inhibition. The inhibition constant K_i_ reflects the intrinsic binding affinity between the inhibitor and enzyme; a lower value indicates stronger binding between the inhibitor and enzyme and higher inhibitory activity. A subplot analysis of the inhibition constants of the three peptides against ACE showed that the K_i_ values for MVPFLQPK, VPFLQPKVM, and QKWKFL were 391.1 ± 1.283, 441.8 ± 2.8293 and 506.8 ± 1.5936 μmol/L, respectively. The binding strengths order was MVPFLQPK > VPFLQPKVM > QKWKFL, which corresponds to the results of the ACE-inhibition assays. VPFLQPKVM exhibited mixed inhibition, with an inhibition constant K_i_ for free ACE (Figure 3B, slopes from the inhibition constant secondary plots) and an inhibition constant K_iS_ for the ACE–substrate complex (Figure 3B, intercept from the inhibition constant secondary plots) of 441.8 ± 2.8293 μmol/L and 1102.9 ± 3.6143 μmol/L, respectively. The affinity for free ACE was significantly higher than that for the ACE–substrate complex, indicating that the inhibitory action of VPFLQPKVM is primarily due to competitive binding. Similar studies by Li et al. [31] employed a combined ultrafiltration and RP-HPLC method to isolate bovine whey protein hydrolysates, identifying four potential ACE-inhibitory peptides (PQVSTPTL, MPGP, PMHIR, PPLT), with IC_50_ values of 86 ± 8, 179 ± 4, 90 ± 6, and 168 ± 4 μmol/L, respectively. Lineweaver–Burk plots indicated that PQVSTPTL exhibited competitive ACE inhibition. PMHIR and PPLT exhibited inhibition patterns similar to that of PQVSTPTL (competitive inhibition), whereas MPGP exhibited a mixed competitive inhibition pattern. The mixed competitive inhibition pattern indicates that MPGP can interact with the allosteric site of ACE. Additionally, the reported soybean protein-derived ACE-inhibitory peptide NDRP was identified as a non-competitive inhibitor, with an IC_50_ of 148.28 ± 9.83 µg/mL [32], which is consistent with the similar inhibitory characteristics exhibited by the peptides VPFLQPKVM and MVPFLQPK in our study, suggesting that peptides bind not only to the active site but also to non-active sites. Binding to the active site directly affects ACE activity, whereas binding at non-active sites may inhibit ACE–substrate interactions through steric hindrance, thereby influencing the enzyme’s maximum reaction rate and substrate affinity. These findings reveal the diversity and complexity of peptide-mediated ACE inhibition.

### 3.3. Molecular Docking

**Figure 4:**
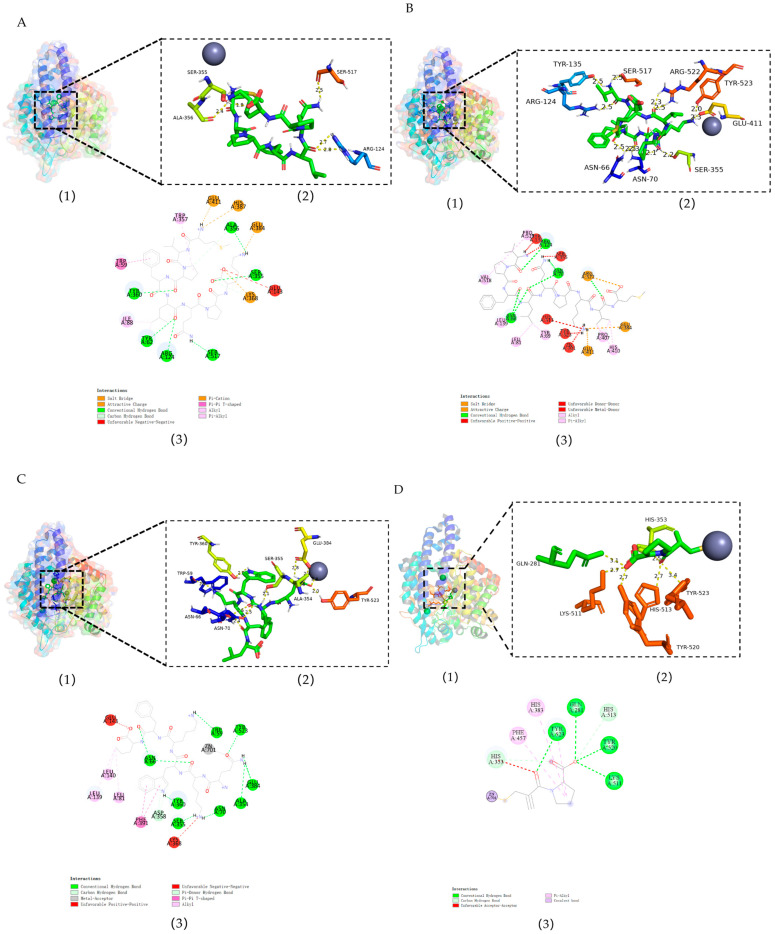
Molecular docking analysis of peptides with ACE. Note: (**A**) molecular docking analysis of peptide MVPFLQPK with ACE; (**B**) molecular docking analysis of peptide VPFLQPKVM with ACE; (**C**) molecular docking analysis of peptide QKWKFL with ACE; (**D**) molecular docking analysis of captopril with ACE (PDB: 1uzf). (**A1**,**B1**,**C1**) show the best-ranked docking modes of peptides with ACE (PDB: 1O8A); (**A2**,**B2**,**C2**) show the general overview of the best docking modes of peptides; (**A3**,**B3**,**C3**) show the 2D protein–ligand interaction diagram; (**D1**) shows the best-ranked docking modes of captopril with ACE (PDB: 1uzf); (**D2**) shows the general overview of the best docking modes of captopril; (**D3**) shows the 2D protein–ligand interaction diagram.

Molecular docking technology can precisely elucidate the molecular recognition process between peptides and ACE residues, directly determining the degree of biological activity exhibited by ACE-inhibitory peptides during their binding to the active site of the enzyme [33]. The S_1_, S_1_′, and S_2_ regions constitute the primary distribution sites for ACE-inhibitory peptides. Ala354, Glu384, and Tyr523 collectively form the critical recognition interface of the S_1_ site. The function of the S_1_′ site relies on the coordinated action of six key residues, including Gln281, His353, Lys511, His513, and Tyr520. The S_2_ site features Glu162 as its characteristic recognition element [34]. As shown in Figure 4, all three peptides entered the active cavity of the ACE receptor. Among these, QKWKFL forms 11 hydrogen bonds with nine amino acid residues, including Ala354, Glu384, and Zn701, establishing a metal–receptor interaction with Zn701. MVPFLQPK can form five hydrogen bonds with four amino acid residues, including Ser355, Ala356 and Ser517, and establishes electrostatic interactions with Glu384, His387, Glu411, and others. The peptide VPFLQPKVM in this study formed 12 hydrogen bonds with nine amino acid residues, including Glu411 and Tyr523. These findings were consistent with the research by Lan Xiong diao et al., who identified an ACE-inhibitory peptide GMKCAF from the long-bodied snake mullet (Sauridaelongata). Molecular docking studies revealed that the peptide interacts with ACE through four hydrogen bond interactions with the amino acid residues Ser480, Ser481, Ala320, and Arg486 [35]. In a similar study, Y. Dong et al. obtained a novel ACE-inhibitory peptide of VGLFPSRSF from tilapia skin hydrolysate. Molecular docking results showed that VGLFPSRSF formed hydrogen bonds with Asn70, His387, Arg522, Glu376, and Asp377 [36].

In this study, the structure of the captopril–human ACE complex (PDB ID: 1UZF) was also investigated, as shown in Figure 4D. It can be seen that captopril forms a stable interaction with Zn701 at the ACE active site, which is a key factor in its potent inhibitory activity. Furthermore, captopril formed seven hydrogen bonds with key active site residues (including Gln281, His353, Lys511, His513, Tyr520, and Tyr523), indicating that captopril binds tightly to the S1, S1′, and S2′ active pockets of ACE. Furthermore, the proline group of captopril formed strong π-π stacking interactions with Phe457 and Tyr523, further enhancing the binding stability within the hydrophobic pocket [37,38]. Thus, it was revealed that captopril can potently inhibit ACE activity, with inhibitory activity significantly higher than that of the three food-derived ACE-inhibiting peptides, consistent with previous ACE inhibition rate assay results. Typically, peptides inhibit ACE activity by forming hydrogen bonds with key amino acid residues, thereby preventing the enzyme from performing its physiological function. Furthermore, the presence of proline enhances the binding stability of the peptide to ACE via hydrophobic interactions, thereby increasing the peptide’s inhibitory activity [39]. Among the three peptides, QKWKFL lacked a proline group, resulting in weaker hydrophobic interactions with ACE and lower overall binding stability. This may explain its weaker inhibitory activity compared to that of the other two peptides. The peptide VPFLQPKVM forms 12 hydrogen bonds with nine amino acid residues, including Glu411 and Tyr523, which allows it to bind stably to the S1 active site of ACE. Simultaneously, the proline groups in its sequence structure form hydrophobic interactions with the ACE active site, enhancing the stability of its binding to ACE. This may explain why the long-chain VPFLQPKVM exhibits a stronger inhibitory activity than the short-chain QKWKFL. The peptide MVPFLQPK formed five hydrogen bonds with four amino acid residues, including Ser355, Ala356, and Ser517, forming five hydrogen bonds. It can form electrostatic interactions with amino acid residues, such as Glu411 in the S1 pocket, and its *C*-terminal proline residue forms a more stable π-π stacking interaction with aromatic residues in the S2′ pocket. This may explain why its inhibitory activity is stronger than that of the other two peptides. This study also observed that these peptides formed hydrophobic interactions with multiple amino acid residues, enabling them to effectively occupy or block the active site of the enzyme. This prevents ACE from interacting with its natural substrate and disrupts the catalytic process, thereby inhibiting its activity.

### 3.4. Molecular Dynamics

Molecular dynamics simulations enable continuous observation of affinity and stability during ACE–ACEIP binding [37,39,40,41]. Therefore, 100-nanosecond molecular dynamics simulations based on the molecular docking results were conducted to explore the synergistic effects of the peptides on ACE inhibition.

**Figure 5:**
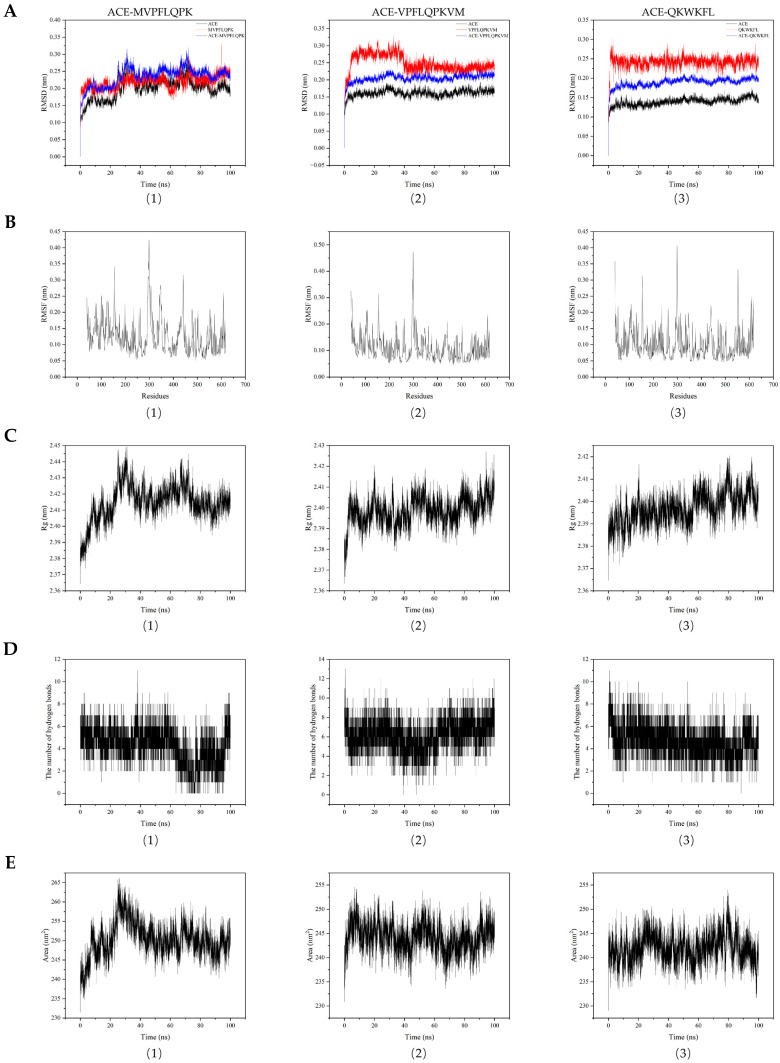
Molecular dynamics simulation results. Note: (**A**) RMSD curves for ACE, peptides, and ACE–peptide complexes; (**B**) RMSF curves for ACE–peptide complexes; (**C**) Rg curves for ACE–peptide complexes; (**D**) hydrogen bond count variation curves for ACE–peptide complexes; (**E**) SASA curves for ACE–peptide complexes. Different numbers represent different treatment groups. (1) ACE–MVPFLQPK, (2) ACE–VPFLQPKVM, (3) ACE–QKWKFL.

The root mean square deviation (RMSD) was used to evaluate conformational changes in the ACE–peptide complexes by comparing the simulated structure with the initial crystal structure. The RMSD values positively correlated with complex stability, with lower average RMSD values indicating higher stability [42]. As shown in Figure 5(A1–A3), the RMSD curve for ACE–QKWKFL reached a plateau during the 10–100 ns time interval, with fluctuations consistently around 0.19 nm. The RMSD curve for ACE–VPFLQPKVM stabilized within the 30–100 ns time frame, fluctuating at approximately 0.21 nm. Similarly, the RMSD curve for ACE–MVPFLQPK stabilized between 30 and 100 ns, fluctuating around 0.25 nm. Among these, the ACE–QKWKFL complex exhibited a smaller RMSD fluctuation range, indicating tighter and more stable binding. The ACE–VPFLQPKVM and ACE–MVPFLQPK complexes reached stability slightly later with larger fluctuation ranges, suggesting that their binding to ACE was relatively less tight and stable than that of the preceding complexes. Related literature indicates that short-chain peptides generally bind more readily to ACE and exhibit greater stability. Moreover, the inhibitory activity of a peptide also depends on its ability to enter the active site and interact with key residues [43,44]. Molecular docking results showed that QKWKFL formed 11 hydrogen bonds with ACE, involving Ala354 and Glu384, and formed a metal-coordination bond with Zn701. Extensive hydrogen bonding and surface electrostatic interactions result in minimal overall conformational shifts and a more stable RMSD in molecular dynamics simulations. However, this peptide primarily binds to the surface region of ACE, and although the binding is stable, it fails to effectively occupy key active sites, such as the hydrophobic S1′ pocket. QKWKFL primarily binds to the protein surface and cannot efficiently occupy key active sites, resulting in weaker inhibition and a higher IC_50_. In contrast, MVPFLQPK penetrated deeply into the hydrophobic S1′ pocket, forming strong interactions with the catalytic residues, thereby reducing ACE activity.

The root mean square fluctuation (RMSF) represents the amplitude of fluctuations of a single amino acid residue deviating from its initial position throughout the simulated system over time T, providing a quantitative representation of the dynamic behavior within a molecule. As shown in Figure 5(B1–B3), the RMSF values for most residues in ACE–QKWKFL range between 0.05–0.20 nm and 0.30–0.35 nm. The overall fluctuations were small, indicating a relatively stable binding state. For ACE–VPFLQPKVM and ACE–MVPFLQPK, most residues exhibited RMSF values within 0.05–0.20 nm, with some residues reaching approximately 0.40 nm. This indicates that their binding to ACE is relatively less tight and stable than that of the preceding complexes. The radius of gyration (Rg) describes the root mean square distance of all atoms or mass centers in a molecule relative to its center of mass [41,45,46]. It reflects the overall shape and compactness of the molecule, with smaller Rg values typically indicating a more compact conformation and larger Rg values suggesting a looser molecular structure. As shown in Figure 5(C1–C3), the Rg curves of ACE after binding to the peptides are depicted. The Rg of ACE–QKWKFL ranged from 2.36 to 2.42 nm, exhibiting relatively minor variations throughout the simulation process and demonstrating excellent structural stability. For ACE–VPFLQPKVM and ACE–MVPFLQPK, the Rg values ranged from 2.36 to 2.45 nm, exhibiting a slightly larger variation. This suggested that these two complexes may have undergone structural relaxation or rearrangement during the simulation but remained generally stable.

In order to further investigate the conformational changes that occur when small molecules bind to proteins, the hydrogen bonds formed between the small molecules and the protein–ligand complexes during a 100 ns molecular simulation were analyzed. As shown in Figure 5(D1–D3), the number of intermolecular hydrogen bonds formed between the three peptides and ACE fluctuated between six and fifteen during the 100 ns simulation. Among these, the ACE–QKWKFL and ACE–MVPFLQPK complexes exhibited significant fluctuations in the number of hydrogen bonds during the simulation, indicating frequent formation and breaking of hydrogen bonds. This suggested that the ACE–peptide docking complexes may induce local structural rearrangements in the protein, leading to an increase or decrease in the number of hydrogen bonds in specific regions. These dynamic changes in hydrogen bonding indicated that the ACE–peptide complexes underwent local structural adjustments to optimize intermolecular interactions. Based on the results of RMSD and Rg, the overall conformational state of the ACE–QKWKFL and ACE–MVPFLQPK complexes remained stable throughout the simulation, with no significant global structural rearrangements or unfolding observed.

The solution-accessible surface area (SASA) measures the surface area of a molecule interacting with a solvent, reflecting the extent of its exposure to the solvent. It provides insights into the solubility, stability, and functionality of molecules under different environmental conditions. In protein molecules, larger SASA values typically correlate with more exposed domains, whereas smaller SASA values indicate a more compact molecular state [47]. As shown in Figure 5(E1–E3), the SASA value of ACE–QKWKFL gradually decreased during the simulation, indicating that the complex was progressively more compact and stable. In contrast, ACE–VPFLQPKVM and ACE–MVPFLQPK exhibited greater SASA fluctuations, indicating looser and less stable structures. Notably, ACE–MVPFLQPK showed a significant increase in SASA during the mid-simulation period, suggesting substantial conformational changes in the complex during this time frame.

The peptide QKWKFL exhibited the best performance in the molecular dynamics simulations. However, its IC_50_ value was higher than those of the other two peptides, indicating weaker ACE-inhibitory activity. This may be attributed to the core parameters of molecular dynamics (RMSD, RMSF, Rg, and SASA) that reflect the overall conformational fluctuations, rigidity, and compactness of the ACE–peptide complex rather than the peptide’s efficiency in inhibiting ACE catalytic activity. According to the molecular docking results, QKWKFL formed abundant hydrogen bonds with the surface residues of ACE, which locked the overall conformation of ACE and resulted in minimal conformational fluctuations, thereby leading to its superior performance in the simulations. However, these interactions are primarily distributed on the peripheral surface of ACE, and QKWKFL fails to penetrate deeply into ACE’s core catalytic domain, such as the hydrophobic S_1_′ active pocket [43]. In contrast, the peptide MVPFLQPK requires a certain degree of conformational rearrangement to enter the narrow active pocket owing to its longer structure, resulting in greater overall conformational fluctuations of the complex and relatively poorer performance in the molecular dynamics simulations. MVPFLQPK effectively occupies the catalytic center, forms strong electrostatic interactions with key catalytic residues, and blocks substrate binding, thereby exhibiting the strongest ACE-inhibitory activity and the lowest IC_50_ value [46]. These results confirmed that the ACE-inhibitory activity of the peptide segment was related to the overall conformational stability of the complex but primarily depends on effective binding to the catalytic active pocket and interactions with key catalytic residues.

MM/PBSA (Molecular Mechanics/Poisson–Boltzmann Surface Area), which combines molecular mechanics (MM), the Poisson–Boltzmann Equation (PB), and surface area (SA) models, provides a precise means to estimate energy changes during biomolecular complex formation. It accurately assesses the binding energies between ligands and receptors (e.g., proteins and small molecules, proteins, and proteins), including ΔV_DWAALS_, ΔE_elec_, ΔE_GB_, ΔE_surf_, ΔG_gas_, ΔG_solvation_, and ΔTotal. A summary of the binding components in the free energy of binding between the peptides and proteins is presented in Table 2.

To analyze the differences in the inhibitory activity among food-derived ACE-inhibiting peptides, we referred to the MM-PBSA energy analysis data for captopril and ACE reported by Belal et al. [48], as shown in Table 2. Based on the results, the ΔTotal values for the three peptides MVPFLQPK, VPFLQPKVM, and QKWKFL were −51.19 ± 3.00 kcal/mol, −34.50 ± 0.99 kcal/mol, and −34.24 ± 1.20 kcal/mol, respectively, whereas that of captopril was −5.36 ± 2.75 kcal/mol. This indicated that the binding affinities of the three peptides were higher than that of captopril. However, the IC_50_ values measured for the three peptides in the in vitro ACE inhibition assays were significantly higher than that of captopril. This may be due to captopril’s ability to tightly bind to a narrow region of the ACE catalytic core, whereas peptides require multiple binding sites within the ACE active pocket to form extensive van der Waals and electrostatic interactions. Therefore, the total binding energy of captopril was weaker than that of the peptides. Secondly, considering the design intent of captopril, it may directly inactivate ACE catalytic activity by chelating Zn^2+^ via its thiol group, whereas peptides require extensive binding to ACE to achieve competitive inhibition, necessitating a strong overall binding affinity. Furthermore, the MM/PBSA results represented the intrinsic thermodynamic binding stability of the ligand–receptor complex at equilibrium [49]. The ΔTotal reflected the intrinsic affinity at the thermodynamic level, which can be used to explain the surface stability of the ligand–receptor complex but does not reflect the kinetic efficiency of inhibition. Therefore, the inhibition mechanisms of captopril and food-derived ACE-inhibiting peptides are different, and the ΔTotal value is correlated with their inhibitory activity.

There was no significant difference in the total binding free energy between QKWKFL and VPFLQPKVM (*p* > 0.05), indicating that those two peptides possess similar ACE-binding affinities. In terms of energy changes, the presence of proline caused ΔE_elec_ to increase by 2.81 kcal/mol for VPFLQPKVM when compared to QKWKFL, indicating that the electrostatic attraction between the peptide chain and the ACE active site was significantly weakened, reducing the stability of the peptide–ACE complex. The ΔE_GB_ was decreased by 3.31 kcal/mol, indicating that the polar desolvation energy barrier was reduced, making the binding process smoother. The ΔG_gas_ was increased by 3.51 kcal/mol, reflecting a decrease in the van der Waals and electrostatic interactions between the VPFLQPKVM peptide and ACE, which is unfavorable for binding to ACE. The ΔG_solvation_ was decreased by 3.77 kcal/mol, indicating that the thermodynamic resistance of the binding process was reduced. Therefore, the presence of proline enhances the stability of the ACE–peptide complex to some extent, which may explain the reason why the IC_50_ value of VPFLQPKVM was slightly lower than that of QKWKFL. Furthermore, energy analysis indicated that MVPFLQPK exhibited the strongest inhibitory activity; it had significantly stronger van der Waals forces (ΔV_DWAALS_ = −51.09 ± 2.19 kcal/mol) and electrostatic interactions (ΔE_elec_ = −74.85 ± 1.90 kcal/mol) than the other two peptides, resulting in tighter binding to ACE and a lower total binding free energy. This result is consistent with the previous study by Zhang Biying [43,50], suggesting that the lowest in vitro IC_50_ value was related to the strongest ACE-inhibitory activity. The results also reveal distinct molecular-level interaction characteristics among the three ACE–peptide complexes, in which van der Waals and electrostatic interactions are the primary determinants of complex stability. Notably, the MVPFLQPK complex exhibited the strongest binding affinity, suggesting its potential significance in related biological functions or applications.

### 3.5. Network Pharmacology Analysis

**Figure 6:**
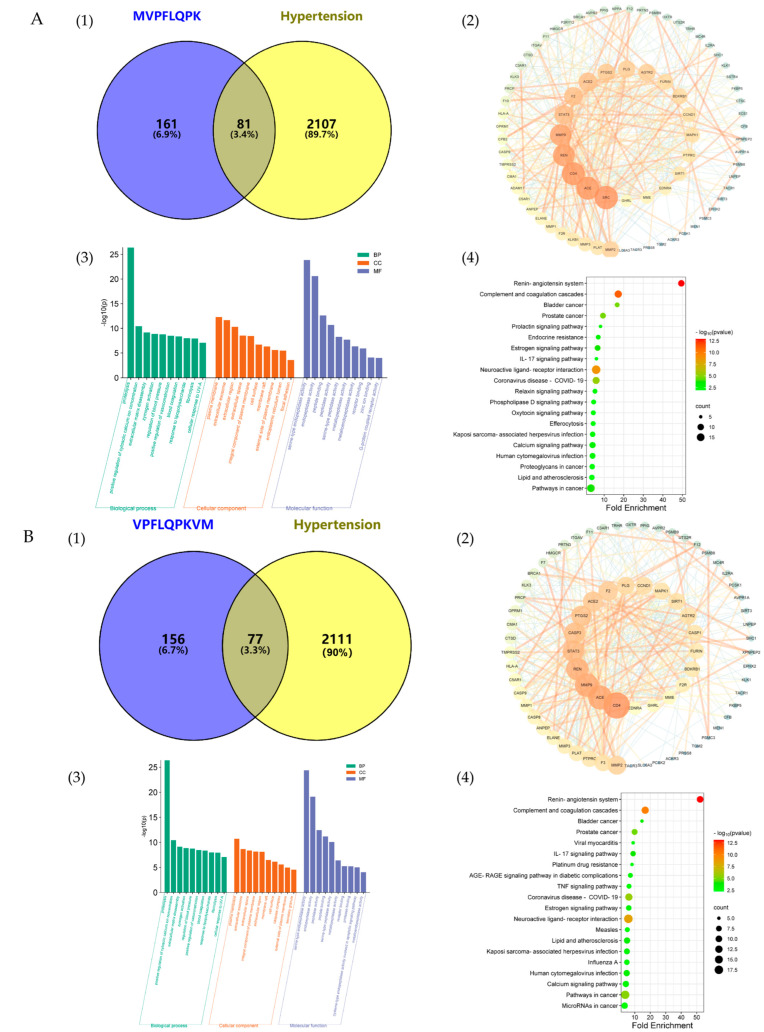

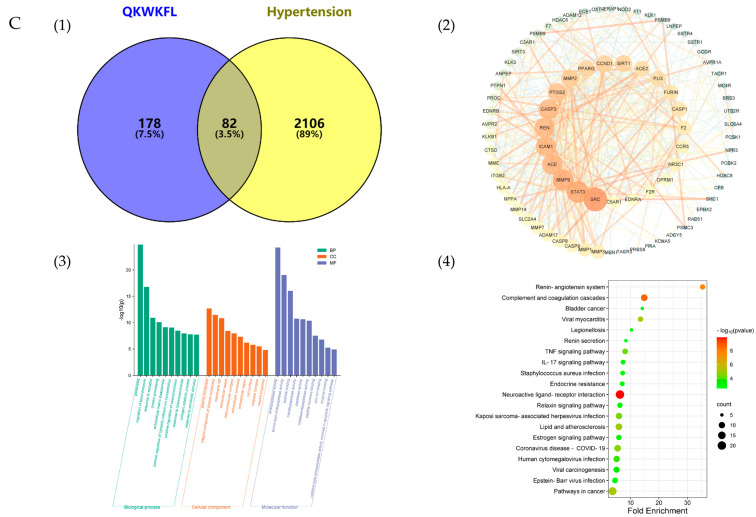
Network pharmacology analysis of the polypeptides. Note: (**A**) network pharmacology analysis results for peptide MVPFLQPK; (**B**) network pharmacology analysis results for peptide VPFLQPKVM; (**C**) network pharmacology analysis results for peptide QKWKFL. From left to right: Venn diagram of potential functional targets (**A1**,**B1**,**C1**); protein–protein interaction network of potential functional targets (**A2**,**B2**,**C2**); GO biological function analysis (**A3**,**B3**,**C3**); KEGG pathway enrichment analysis (**A4**,**B4**,**C4**).

The potential target sites of the three peptides and the results of the network pharmacology analysis are illustrated in Figure 6. Potential overlapping targets were identified by leveraging a comprehensive database of peptides and genes associated with hypertension regulation. As shown in Figure 6(A1,B1,C1), the peptide MVPFLQPK has 242 predicted potential target sites, whereas peptides QKWKFL and VPFLQPKVM each have 233 predicted potential targets. The Venn diagram indicates that MVPFLQPK shares 81 hypertension-related targets, whereas QKWKFL and VPFLQPKVM share 82 and 77 hypertension-related targets, respectively.

The intersecting target data were then uploaded to the STRING database for protein–protein interaction analysis. In protein–protein interaction (PPI) analysis, larger circles indicated higher degree values for target proteins [50,51,52]. The results (Figure 6(A2,B2,C2)) revealed that the potential antihypertensive target PPI network of MVPFLQPK contained 499 target interactions. Among these, 20 key potential targets, including SRC, CD4, ACE, REN, and MMP9, were identified using the following thresholds: closeness > 0.0062, betweenness > 86.1519, and degree > 12. Meanwhile, the QKWKFL potential antihypertensive target PPI network contained 501 target interactions. Among these, 23 key potential targets were identified using the following criteria: closeness > 0.0059, betweenness > 94.5926, and degree > 12, including SRC, STAT3, MMP9, ACE, ICAM1, and REN. The potential antihypertensive target PPI network for VPFLQPKVM contained 470 target interactions, and similarly, 21 key potential targets were identified using the following criteria: closeness > 0.0064, betweenness > 85.5733, and degree > 12, including CD4, ACE, MMP9, REN, STAT3, et al. PPI analysis based on the predicted targets suggested that AKT1, TP53, IL6, TNF, CASP3, and VEGFA were the common core hub targets of the three peptides. These predicted targets were not only associated with blood pressure regulation but also enriched in biological processes, including apoptosis, cell proliferation, angiogenesis, and tissue repair, suggesting that these three peptides may have potential regulatory effects on these physiological processes, which need to be verified by subsequent in vitro and in vivo experiments [53].

The network pharmacology analysis was conducted to explore the potential antihypertensive mechanisms of these three peptides. As shown in Figure 6, Gene Ontology (GO) enrichment analyses (Figure 6(A3,B3,C3)) indicated that the predicted antihypertensive targets of MVPFLQPK, VPFLQPKVM, and QKWKFL were primarily associated with blood pressure regulation, vasoconstriction regulation, and intracellular calcium ion concentration regulation. These findings suggested that the three peptides may potentially interfere with the core processes of blood pressure regulation (including vascular tone regulation) and related signaling pathways (such as calcium signaling pathways); meanwhile, they may influence cellular components, such as the plasma membrane and cell surface, by recognizing or interfering with signals on the cell membrane. Simultaneously, the molecular functions of these three peptides were enriched in peptidase activity, peptide binding, and zinc ion binding, suggesting that they may exert their potential biological functions by interacting with ACE and related zinc-binding peptidases.

KEGG pathway analysis identified key pathways involved in the action of ACE-inhibiting peptides (Figure 6(A4,B4,C4)), where higher orange concentrations within bubbles indicate greater significance and larger bubble areas represent a higher degree of clustering of targets within that pathway [50,54]. These results suggest that MVPFLQPK may exert its potential antihypertensive effects through the renin–angiotensin system, complement system, and coagulation cascade, whereas QKWKFL and VPFLQPKVM may also be involved in the regulation of these pathways [55,56].

In summary, the network pharmacology analysis indicated that these three peptides derived from camel casein (MVPFLQPK, QKWKFL, and VPFLQPKVM) may primarily exert their potential blood pressure-regulating effects by targeting ACE. Additionally, the predicted targets included other blood pressure-related molecules, such as REN, CASP3, and MMP9, which are involved in the regulation of the renin–angiotensin, angiogenesis, and apoptosis pathways. This supported the hypothesis that the antihypertensive effects of these three peptides may involve multi-target and multi-pathway regulation.

### 3.6. Assay for Peptide Cytotoxicity

**Figure 7:**
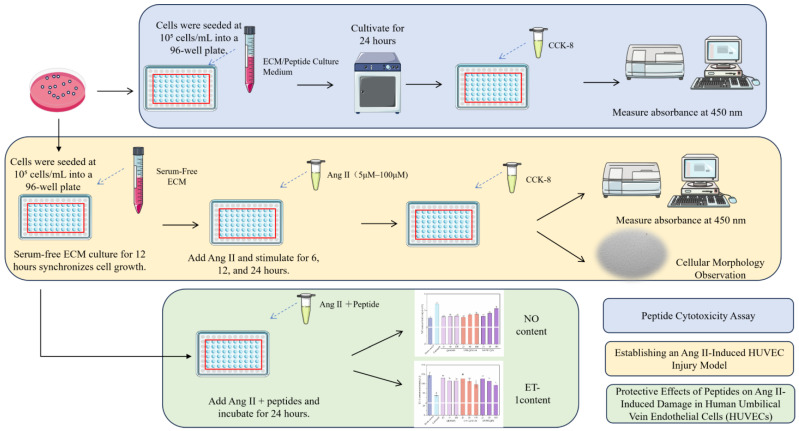
Cell Experiment Flowchart.

Human umbilical vein endothelial cells (HUVECs) are important for studying blood vessel biology and medicine. They help test how different substances affect blood vessel function [57]. ACE-inhibitory peptides can lower blood pressure, improve blood vessel function, reduce inflammation and stress, and keep cells healthy. HUVECs help blood vessels widen and narrow by releasing substances like nitric oxide (NO) and endothelin-1 (ET-1). NO helps relax blood vessels, lowering blood pressure. ET-1 causes blood vessels to tighten. Previous reports have shown that ACE-inhibitory peptides are important for blood vessel health because they keep blood pressure normal and prevent related problems [58]. However, there is limited research on how camel casein ACE-inhibitory peptides affect HUVECs. The cellular experimental workflow depicted in Figure 7 elucidates how camel milk peptides safeguard endothelial cell health by revealing the dynamic changes in nitric oxide (NO) and endothelin-1 (ET-1) levels within the HUVECs cell model upon treatment with three distinct camel milk peptides.

**Figure 8:**
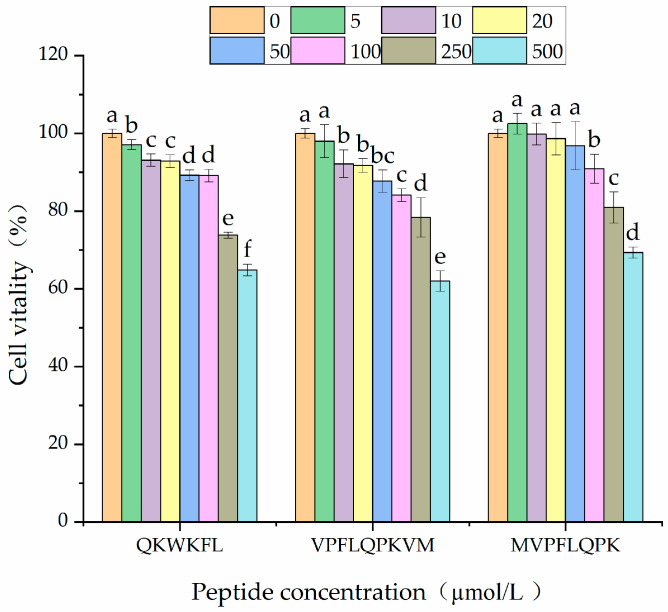
Effects of different peptide concentrations (μmol/L) on the survival rate of human umbilical vein endothelial cells. Different lowercase letters (a–f) on the bars indicate that the difference is significant (*p* < 0.05).

The results of the cytotoxicity assays are shown in Figure 8. The three peptides, QKWKFL, VPFLQPKVM and MVPFLQPK, exhibited significant differences in their effects on cell viability across various concentrations (0, 5, 10, 20, 50, 100, 250, and 500 µmol/L). Within the low concentration range (0–100 µmol/L), all three peptides maintained high levels of cell viability, exceeding 80%. However, at higher concentrations (≥250 µmol/L), the cell viability significantly decreased. At 250 µmol/L, the viability of cells cultured with QKWKFL and VPFLQPKVM fell below 80%, reaching 73.76 ± 2.321% and 78.35 ± 1.563%, respectively. At the highest concentration (500 μmol/L), all peptides exhibited the most pronounced inhibitory effects on cell viability, with viability dropping below 80% across all cell lines. Among them, VPFLQPKVM showed the greatest reduction in cell viability, which fell to 61.81 ± 2.842%. At low concentrations (0–50 μmol/L), this finding aligned with that of Lin Kai et al. [57], who identified a novel ACE-inhibitory peptide of KYIPIQ from yak milk casein and further suggested that treatment with KYIPIQ at a concentration of 100 μmol/L exhibited no cytotoxic effects on HUVECs. In the current study, within an appropriate concentration range, peptides significantly maintained cell viability, with certain peptides, such as VPFLQPKVM and MVPFLQPK, markedly enhancing viability at low concentrations. However, at excessively high concentrations, peptides can exert cytotoxic effects on cells. Therefore, careful control of the peptide concentration is essential for practical applications. Based on the comprehensive experimental results, subsequent experiments were conducted using the three peptides at concentrations of 25, 50, and 100 μmol/L as low-, medium-, and high-dose groups, respectively.

### 3.7. Effects of Peptides on Angiotensin II-Induced Injury Models

#### 3.7.1. Establishment of an Angiotensin II (Ang II) Injury Model

**Figure 9:**
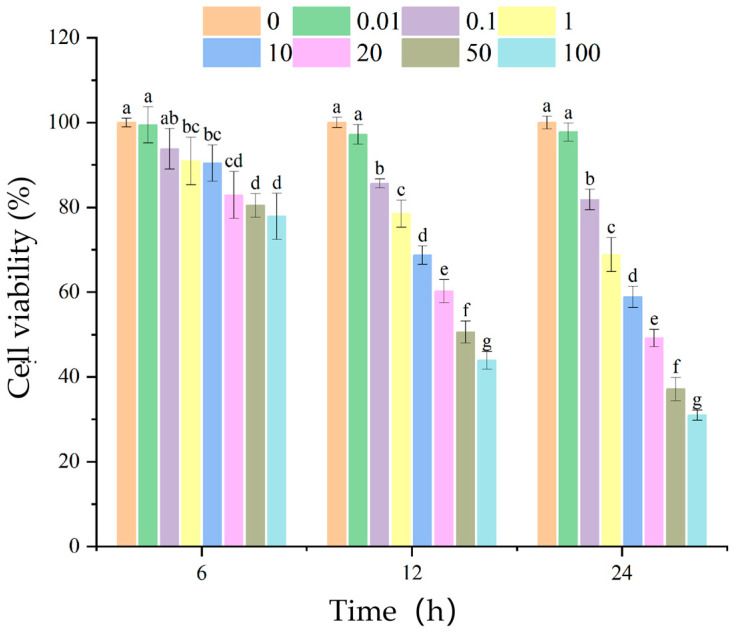
Effects of different concentrations of Ang II on HUVEC viability. Different lowercase letters (a–g) on the bars indicate that the difference is significant (*p* < 0.05).

**Figure 10:**
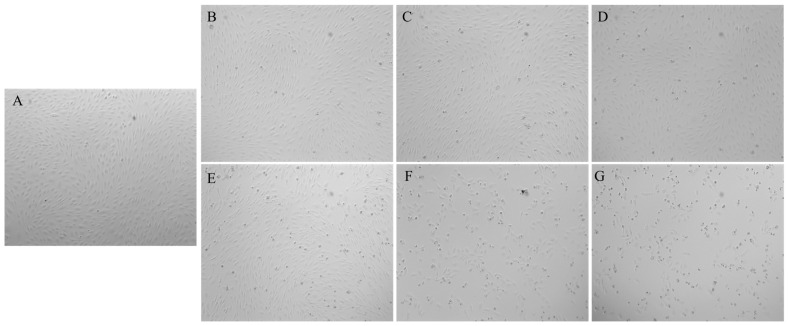
Inverted microscope images (10 × 10) of HUVECs treated with different concentrations of Ang II. Note: (**A**) blank control group; (**B**–**D**) 1μmol/L Ang II treated for 6 h, 12 h, and 24 h, respectively; (**E**–**G**) 100 μmol/L Ang II treated for 6 h, 12 h, and 24 h, respectively.

The potential cytotoxic effects of Ang II on HUVEC viability was assessed. The CCK-8 results demonstrated that the effects of Ang II on HUVEC viability were both concentration and time dependent (Figure 9). Compared to the blank group, the Ang II-treated group exhibited a 68.8 ± 1.122% reduction in cell viability at 1.0 μmol/L after 24 h treatment. Based on combined cell viability and morphological observations, Ang II treatment at 1.0 μmol/L for 24 h was selected as the optimal injury concentration and duration for subsequent experiments. Morphological changes were observed in the cell model. After 24 h of Ang II induction, significant filamentous cell morphology was evident, with a noticeable reduction in cell count, blurred cell boundaries, and loss of three-dimensional structure. At 100.00 μmol/L Ang II incubation for 24 h, the cell count decreased significantly, and severe cell death occurred, rendering this concentration unsuitable for establishing a hypertension-induced injury model (Figure 10). Therefore, 1.0 μmol/L of Ang II was ensured as the optimal concentration for establishing the model.

#### 3.7.2. Effect on NO Release Behaviors

**Figure 11:**
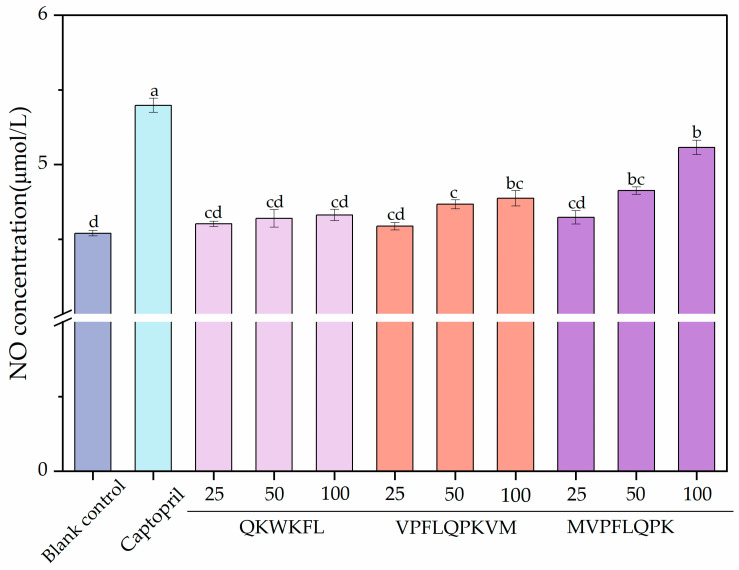
Effects of different peptide concentrations on NO level trends in a cellular injury model. Different lowercase letters (a–d) on the bars indicate that the difference is significant (*p* < 0.05).

**Figure 12:**
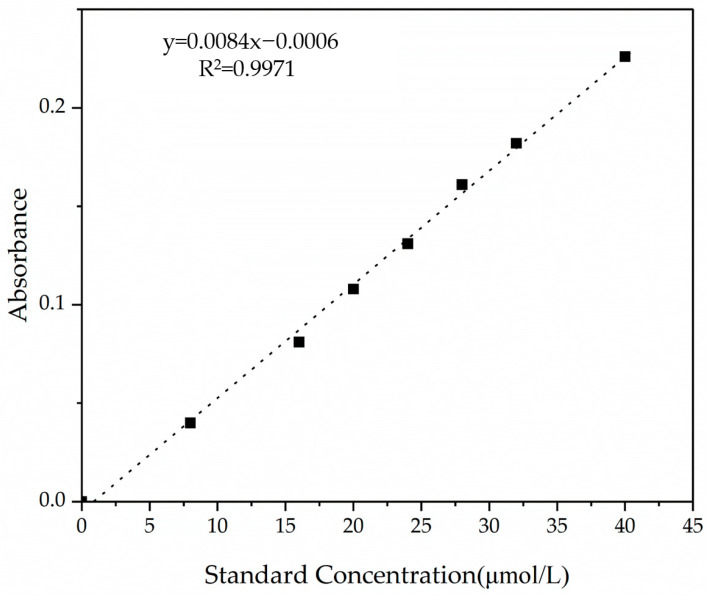
Standard curves for NO content.

The effects of different peptide treatments on NO release by HUVECs are shown in Figure 11. The concentration of NO calculated using the standard curve is shown in Figure 12. Compared with the blank control group, both the positive control and peptide groups showed increased NO release behavior in a dose-dependent manner. Specifically, the MVPFLQPK peptide group showed a significant increase in NO content with increasing peptide concentration. At 100.0 μmol/L, NO production was significantly higher than that of QKWKFL and VPFLQPKVM. Conversely, VPFLQPKVM demonstrated higher NO release than QKWKFL at 50 and 100 μmol/L. This finding was consistent with its stronger in vitro ACE-inhibitory activity. Experimental results indicated that all three camel milk casein ACE-inhibitory peptides significantly enhanced NO release at certain concentrations; the structural features of MVPFLQPK may potentially favor the activation of endothelial nitric oxide synthase (e NOS) or enhanced ACE inhibition. The current data indicated that the specific mechanism by which peptides promote NO release in HUVECs primarily involves the activation of endothelial nitric oxide synthase (e NOS), thereby increasing NO synthesis. Certain peptides can reduce angiotensin II (Ang II)-induced endothelin-1 (ET-1) synthesis and activate eNOS to promote NO production [54,59]. Additionally, these peptides can enhance NO release through alternative pathways, such as the PI3K/Akt/e NOS pathway [60]. On the other aspect, NO participated in both peripheral and central regulation of the cardiovascular system. It may antagonize the effects of angiotensin II on vascular tone, cell growth, and renal sodium excretion while downregulating the synthesis of angiotensin-converting enzyme (ACE) and angiotensin II type 1 receptors [44,59,60].

#### 3.7.3. Effect on ET-1 Release

**Figure 13:**
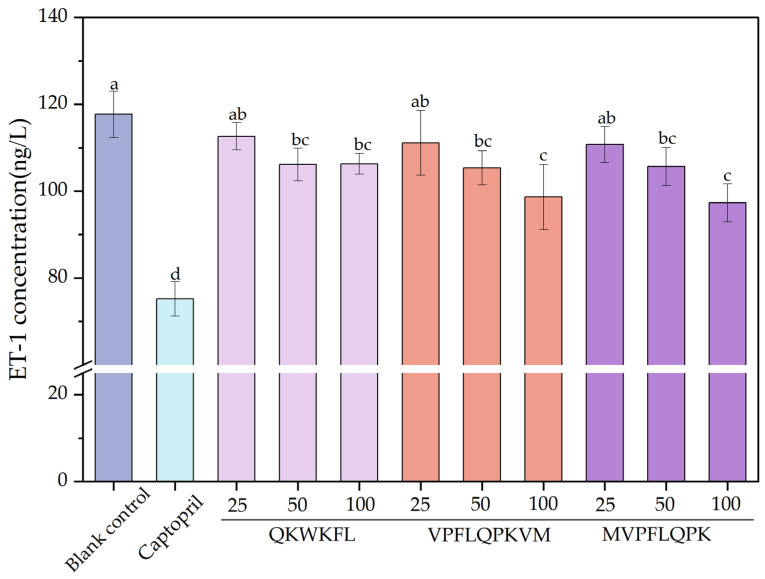
ET-1 secretion in the peptide culture group. Different lowercase letters (a–d) on the bars indicate that the difference is significant (*p* < 0.05).

**Figure 14:**
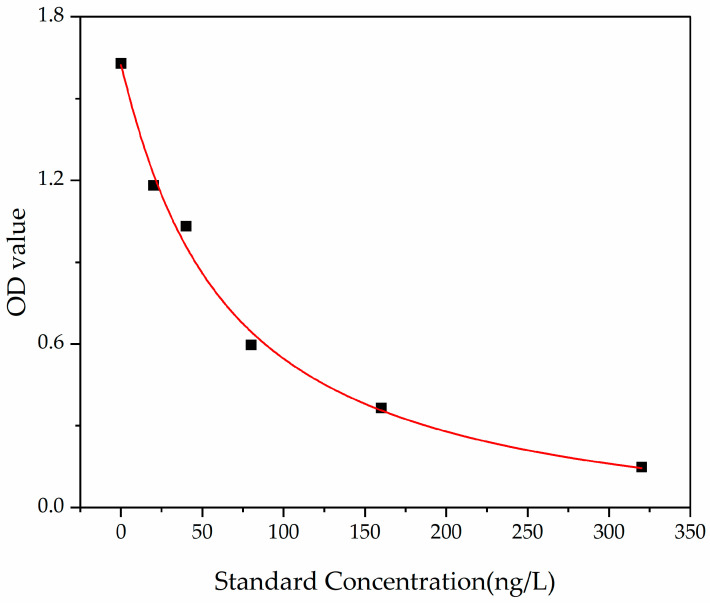
Standard curves for ET-1 content. Note: using ELISA Calc for logistic curve fitting (four-parameter model), the fitted curve is obtained: y = (A − D)/[1 + (x/C)^B^] + D, where A = 1.62366, B = 1.04705, C = 64.03259, D = −0.12967, and r^2^ = 0.99373.

Endothelin-1 (ET-1) is a potent vasoconstrictive peptide with paracrine and local effects. It is the only endothelin produced by the endothelial cells. Under certain pathological conditions, such as hypertension, uremia, and cardiogenic shock, ET-1 levels increase significantly [38]. Figure 13 shows the effects of different peptide treatments on ET-1 release in HUVECs. The concentration of ET-1 was calculated using the standard curve shown in Figure 14. When the peptide concentration was increased from 25 ng/L to 100 ng/L, the ET-1 levels in the MVPFLQPK- and VPFLQPKVM-treated groups decreased by approximately 16.15% and 17.32%, respectively, compared to the blank control group. MVPFLQPK exhibited a slightly stronger ET-1-lowering effect than VPFLQPKVM. Meanwhile, ET-1 release at all QKWKFL concentrations was lower than that of the blank control, showing minimal variation between 50 ng/L and 100 ng/L concentrations, and its ET-1-lowering effect was weaker than that of MVPFLQPK and VPFLQPKVM. The above experimental results indicated that the three casein ACE-inhibitory peptides significantly suppressed ET-1 secretion at certain concentrations. This effect is closely related to the mechanism by which ACE-inhibitory peptides antagonize the Ang II/AT1R signaling pathway. Ang II activates NADPH oxidase to induce ROS production, which, in turn, activates the NF-κB and AP-1 pathways to promote ET-1 transcription [61]. The inhibitory effect of peptides on ET-1 may be related to their dual regulation of ACE activity and the ROS/NF-κ B signaling axis. As a known vasoconstrictor, ET-1 is similar to Ang II, which both can lead to endothelial dysfunction, hypertension, and atherosclerosis [62]. In a similar study, Wang et al. [63] identified two ACE-inhibitory peptides of IVTNWDDMEK and VGPAGPRG from the sea snail *Volutharpa ampullacea perryi*. Both peptides significantly increased NO production in a dose-dependent manner and inhibited ET-1 secretion. Meanwhile, ET-1 mRNA expression also exhibited a similar trend.

In this study, three casein-derived ACE-inhibitory peptides demonstrated a significant capacity to promote NO release and inhibit ET-1 secretion at certain concentrations. These peptides induce vasodilation by enhancing NO secretion and antagonizing excessive vasoconstriction through their inhibitory effect on ET-1 synthesis. This finding aligns with the existing literature, indicating that ACE-inhibitory peptides from diverse sources have the potential to regulate vascular function. This further supports the proposed mechanism of action of ACE-inhibitory peptides in maintaining vascular health.

## 4. Conclusions

This study investigated the three ACE-inhibitory peptides (QKWKFL, VPFLQPKVM and MVPFLQPK) obtained from enzymatically hydrolyzed camel casein components. The results indicated that the IC_50_ values for the ACE-inhibitory peptides MVPFLQPK, VPFLQPKVM, and QKWKFL were 277.1 μmol/L, 396.9 μmol/L, and 486.9 μmol/L, respectively. Enzyme inhibition kinetic analysis revealed that peptides QKWKFL and VPFLQPKVM exhibited non-competitive inhibition within the mixed inhibition category, whereas MVPFLQPK demonstrated a non-competitive-antagonistic inhibition pattern within the same category. Molecular docking results indicated that all three peptides formed hydrogen bond interactions with ACE. Specifically, QKWKFL and VPFLQPKVM establish hydrogen bonds with key amino acid residues in the ACE active site, thereby obstructing ACE contact with its natural substrate or its catalytic process, thus inhibiting enzyme activity. Molecular dynamics simulations indicated that the three peptides and ACE protein docking complexes achieved stable states during the simulation process and exhibited strong binding forces. The binding free energies of the three peptides of QKWKFL, VPFLQPKVM, and MVPFLQPK were −34.24 ± 1.20, −34.50 ± 0.99, and −51.19 ± 3.00 kcal/mol, respectively. Hydrogen bonds, van der Waals interactions, electrostatic interactions, and nonpolar solvation energy were identified as the primary factors determining complex stability as well as for the inhibitory activity. Network pharmacology analysis revealed that the three novel ACE-inhibitory peptides exert their effects by targeting ACE, REN (Renin), SRC (SRC Proto Oncogene, Non-Receptor Tyrosine Kinase), and MMP9 (Matrix Metallopeptidase 9), which play key roles in the blood pressure-lowering process. These antihypertensive targets are most enriched on the renin–angiotensin system, renin secretion, complement and coagulation cascades, neuroactive ligand–receptor interaction, lipid and atherosclerosis, and calcium signaling pathway. This suggested that the newly identified ACE-inhibitory peptides may exert their effects through multiple pathways by synergistically regulating blood pressure. All three peptides exhibited no potential cytotoxicity, significantly promoted NO release, inhibited ET-1 secretion, and exerted vasodilatory and anti-inflammatory effects. This demonstrated that the peptides provide certain protective effects against Ang II-induced HUVEC injury and have potential antihypertensive effects. These findings provide foundational experimental data and research references for the subsequent in vivo validation of the antihypertensive activity of camel milk peptides and the early-stage development of related functional products in the future.

## Figures and Tables

**Table 1:** Structural information of the three peptides.

Constituent	Protein	Position	Start	AA Sequence	End	Mass	Length
JF1	Alpha-S2-casein	82~87	Y	QKWKFL	Q	831.4643	6
JF1-3	Beta-casein	101~109	M	VPFLQPKVM	D	1057.5994	9
JM1	Beta-casein	100~107	V	MVPFLQPK	V	958.5310	8

Note: JF1: <1 kDa fraction of camel milk protein hydrolysate by alkaline protease + flavourzyme; JF1-3: 1–3 kDa fraction of the same hydrolysate; JM1: <1 kDa fraction of camel milk protein hydrolysate by alkaline protease + papain. AA: Amino Acid.

**Table 2:** Results of MM/PBSA calculations for ACE–polypeptide complexes.

Contribution Components	MVPFLQPK	VPFLQPKVM	QKWKFL	Captopril
ΔV_DWAALS_	−51.09 ± 2.19	−34.80 ± 0.11	−35.49 ± 1.17	−21.60 ± 1.93
ΔE_elec_	−74.85 ± 1.90	−31.32 ± 0.82	−34.13 ± 0.22	−10.27 ± 1.73
ΔE_GB_	82.25 ± 0.78	36.64 ± 0.53	39.95 ± 0.12	11.74 ± 0.91
ΔE_surf_	−7.50 ± 0.12	−5.03 ± 0.07	−4.75 ± 0.01	−17.88 ± 0.18
ΔG_gas_	−125.94 ± 2.90	−66.11 ± 0.83	−69.62 ± 1.19	−31.87 ± 2.59
ΔG_solvation_	74.75 ± 0.79	31.61 ± 0.54	35.38 ± 0.12	26.52 ± 0.93
ΔTotal	−51.19 ± 3.00	−34.50 ± 0.99	−34.24 ± 1.20	−5.36 ± 2.75

Note: ΔV_DWAALS_: Van der Waals energy change; ΔE_elec_: electrostatic energy change; ΔE_GB_: generalized Born energy change; ΔE_surf_: surface energy change; ΔG_gas_: gas-phase free energy change; ΔG_solvation_: solvation free energy change; ΔTotal: total free energy change.

## Data Availability

The original contributions presented in this study are included in the article; further inquiries can be directed to the corresponding author.

## Abbreviations

ACE	Angiotensin-Converting Enzyme
ACN	Acetonitrile
Akt	Protein Kinase B
Ang I	Angiotensin I
Ang II	Angiotensin II
AP-1	Activator Protein-1
AT1R	Angiotensin II Type 1 Receptor
CCK-8	Cell Counting Kit-8
DAVID	Database for Annotation, Visualization and Integrated Discovery
Dis Ge NET	Disease-Gene Network
ECM	Endothelial Cell Medium
ΔE_elec_	Electrostatic Energy Change
ΔE_GB_	Generalized Born Energy Change
ELISA	Enzyme-Linked Immunosorbent Assay
eNOS	Endothelial Nitric Oxide Synthase
ΔE_surf_	Surface Energy Change
ET-1	Endothelin-1
ΔG_gas_	Gas-phase Free Energy Change
ΔG_solvation_	Solvation Free Energy Change
GO	Gene Ontology
HA	Hippuric Acid
HHL	Hippuryl-His-Leu
HPLC	High Performance Liquid Chromatography
HUVECs	Human Umbilical Vein Endothelial Cells
IC_50_	Half Maximal Inhibitory Concentration
KEGG	Kyoto Encyclopedia of Genes and Genomes
KKS	Kallikrein–Kinin System
MD	Molecular Dynamics
MMP9	Matrix Metallopeptidase 9
MM/PBSA	Molecular Mechanics/Poisson–Boltzmann Surface Area
NF-κB	Nuclear Factor Kappa-B
NO	Nitric Oxide
NPT	Isothermal-Isobaric Ensemble
NVT	Isothermal and Isovolumetric Ensemble
OMIM	Online Mendelian Inheritance in Man
PI3K	Phosphatidylinositol 3-Kinase
PDB	Protein Data Bank
PPI	Protein–Protein Interaction
RAS	Renin–Angiotensin System
REN	Renin
Rg	Radius of Gyration
RMSD	Root Mean Square Deviation
RMSF	Root Mean Square Fluctuation
RP-HPLC	Reversed-Phase High Performance Liquid Chromatography
ROS	Reactive Oxygen Species
SASA	Solvent-Accessible Surface Area
SEA	Similarity Ensemble Approach
SRC	SRC Proto Oncogene, Non-Receptor Tyrosine Kinase
STRING	Search Tool for the Retrieval of Interacting Genes/Proteins
TFA	Trifluoroacetic Acid
ΔV_DWAALS_	Van der Waals Energy Change
WHO	World Health Organization
ΔTotal	Total Free Energy Change
